# Evolution of Food Classification Systems and Assessing Diet Quality

**DOI:** 10.1016/j.advnut.2026.100596

**Published:** 2026-02-09

**Authors:** Andrea Krenek, Christopher D Gardner

**Affiliations:** Stanford Prevention Research Center, Department of Medicine, School of Medicine, Stanford University, Palo Alto, CA, United States

## Evaluating Dietary Quality

Diet quality in standard American diet patterns consistently remains low [1]. Evaluation of dietary quality serves a range of essential purposes in research, clinical, and public health settings. Beyond investigative contexts, such as examining effects of a nutrition intervention or exposed cohort population on disease risks, diet quality assessment provides monitoring parameters for medical nutrition therapy as well as informs policies and food assistance programs, clinical practice guidelines, and nutrition programming initiatives. In general, evaluation of overall dietary quality involves combining the assessment of 2 domains: *1*) what to emphasize or include, and *2*) what to limit or avoid.

Historical approaches to generate recommendations have included nutrients, foods, and patterns. In recent years, tools for analyzing diet quality have expanded, and some now address food formulation and processing classification (FF&PC) systems [2,3]. These metrics may provide novel, valuable insight into nutrition quality, although they currently present areas of controversy and nuance that are unresolved in most classification systems. In efforts to partly address these challenges, Bernstein et al. [4] conceptualized 9 principles to guide scientists in the development and application of FF&PC systems. These principles expand upon considerations across traditional diet quality indicators, which have historically varied in focus.

## Adding Formulation and Processing Metrics to Diet Quality Indicators

Indicators of diet quality have spanned paradigms and metrics that reflected the most prominent concerns overshadowing nutrition science over time, from isolated nutrients with the discovery of vitamins during the first half of the 20th century progressing to foods and then diet pattern identification for chronic disease prevention [5].

Broadly and historically outlined in Table 1, each subsequent approach has aimed to address lacking components from prior paradigms, although none comprehensively capture all necessary considerations. The United States Dietary Reference Intakes provide recommendations for amounts of specific nutrients, such as vitamins and minerals, to consume and upper limits not to exceed. The Dietary Guidelines for Americans (DGA) aim to translate nutrient recommendations into defined servings of foods. Patterns have been generated from the DGA or adapted for disease-specific guidance by organizations including the American Heart Association [6].

**TABLE 1:** Summary evolution of diet quality indicators

Paradigm	Key concept	Example metrics	Limitations
Nutrient reductionism	Individual nutrient adequacy	Dietary Reference Intakes (EAR, RDA, AI, UL, EER, and AMDR), Mean Adequacy Ratio, and Nutrient Rich Foods Index	Ignore food synergies and diet context
Pattern-based assessment	Overall eating pattern predicts health outcomes	Guideline-based: Healthy Eating Index and Alternative Healthy Eating Index Specific patterns: Mediterranean Diet Score, aMed Diet, KIDMED, DASH Score, MIND diet score, Whole Plant Food Density, PDI, Healthful/Unhealthful PDI, and A Priori Diet Quality Score	Ability to distinguish quality within food groups and matrix
Mechanistic proxies	Diet quality captured through specific biological pathways	Inflammatory: Dietary Inflammatory Potential and Empirical Dietary Inflammatory Pattern Glycemic: Glycemic Index and Glycemic Load Oxidative/acid-base: Dietary Total Antioxidant Capacity and Potential Renal Acid Load	Focus on single mechanisms lack consideration of processing and bioavailability
Processing-based classification	Degree/type of food matrix industrial processing alters health effects independent from nutrients	NOVA, IARC/EPIC, IFPRI, IFIC, UNC, and Siga score	Standardization, mechanistic understanding, and flawed equation of level of processing with healthfulness
Integrated systems (emerging)	Multi-dimensional quality (nutrients, patterns, processing, and sustainability)	Planetary Health Diet Index, EAT-Lancet index, and World Index for Sustainability and Health	Standardization and validation

Abbreviations: AI, adequate intake; AMDR, average macronutrient distribution range; aMed Diet, alternative Mediterranean diet score; DASH, Dietary Approaches to Stop Hypertension; EAR, estimated average requirement; EER, estimated energy requirements; IACR/EPIC, International Agency for Research on Cancer/European Prospective Investigation into Cancer and Nutrition; IFIC, International Food Information Council; IFPRI, International Food Policy Research Institute; KIDMED, Mediterranean Diet Quality Index for children and adolescents; MIND, Mediterranean-DASH Intervention for Neurodegenerative Delay; PDI, plant-based diet index; RDA, recommended dietary allowance; UL, tolerable upper intake level; UNC, University of North Carolina at Chapel Hill.

Progressing from a reductionist view of considering individual nutrients contained in foods separately from the foods themselves, pattern-based and mechanistic indicators have largely dominated diet quality evaluation and continue to provide valuable insight. Pattern-based assessment often applies diet quality indices (DQIs), tools used to evaluate adherence to and predefined quality of a dietary pattern, i.e., the extent to which intake aligns with a specified pattern. Over 100 DQIs have been created across populations, ages, conditions [5,7], and country-specific adaptations [[6], [7], [8], [9]]. These vary in most appropriate method of diet assessment or biomarker data collection needed to be able to calculate the DQI score (e.g., food frequency questionnaire compared with food recall compared with blood samples). Notably, DQIs are only as meaningful as the measure of adherence to which the indicators reflect and generally lack the ability to evaluate the level of refinement and industrial processing that may impact the food matrix or ingredient composition.

Even more rapidly than methods of diet quality assessment have evolved, food production and processing techniques have significantly transformed food systems since vitamin discovery with impacts on cost, taste, convenience, and shelf life. Well known systems for evaluation of ultra-processed foods were not introduced until 2006–2018, with a slight lag in gaining traction in nutrition research [3].

Highly processed foods as classified by FF&PC systems are often considered choices to limit due to poor diet quality, traditionally determined by excess saturated fat, added sugar, sodium, and empty calories. However, multiple exceptions have been identified as several relatively higher quality, nutrient-dense items associated with reduced disease risks may also fall into ultra-processed food categories (e.g., tofu, low-sodium canned beans, 100% whole grain bread). In some cases, the opposite is true as there are minimally processed foods that are recommended by DQIs as items to limit (e.g., butter, red meat) [10].

With these limitations and the growing consensus that the amount of formulation and processing that is involved in contributing adversely rates of diet-related chronic diseases in the United States, it is important to be able to appropriately evaluate processing of foods.

## FF&PC Systems

Many aspects of food formulation and processing can have positive impacts on the food supply, including improved food safety, reduced costs, extended shelf life, and preservation of nutritional, functional, and sensory qualities. Nevertheless, balancing advantages with negative impacts of excessive processing remains challenging, along with evaluation of these factors through classification systems. The principles proposed by Bernstein et al. appear well-intentioned, although it may be worth noting if it is possible to achieve this with exclusivity of FF&PC approaches, without components of other forms of diet quality indicators previously described or to be developed.

Furthermore, although rigorous data is essential, establishing causality requirements may require a level of leniency to maintain a metric for evaluating heavily processed foods to eventually support a food supply and intake of higher quality. For example, Generally Recognized as Safe ingredients meet safety standards with reasonable certainty of no harm, whereas establishing causality is likely unfeasible within research constraints.

The authors note the principles are not designed for development of new systems, although it may be important to determine if the proposed guidance will be more of a barrier or a facilitator for ultimately reducing foods of poor dietary quality. From a broad viewpoint, working toward development of comprehensive, integrated diet quality evaluation may be more beneficial for improving health outcomes.

## Funding

The NIH T32 postdoctoral funding supporting Dr. Krenek:NIH T32 HL161270.

## Conflict of interest

The authors report no conflicts of interest.
